# Evaluation of vitrification for cryopreservation of teeth

**DOI:** 10.5051/jpis.2010.40.3.111

**Published:** 2010-06-25

**Authors:** Surangi C. Dissanayake, Zhong-Min Che, Seong-Ho Choi, Seung-Jong Lee, Jin Kim

**Affiliations:** 1 Oral Cancer Research Institute, Department of Oral Pathology, Yonsei University College of Dentistry, Seoul, Korea.; 2 Department of Periodontology, Yonsei University College of Dentistry, Seoul, Korea.; 3 Department of Conservative Dentistry, Yonsei University College of Dentistry, Seoul, Korea.

**Keywords:** Cryopreservation, Periodontal ligament, Tissue banks

## Abstract

**Purpose:**

The aim of this study was to investigate whether vitrification in the cryopreservation of periodontal ligament (PDL) cells could be useful for tooth banking.

**Methods:**

In step 1, primary cultured human PDL cells were cryopreserved in 100% conventional cryopreservation media and 100% vitrification media (ESF40 media) in different temperatures for 2 weeks. In step 2, a series of modified vitrification formulae named T1 (75% vitrification media + 25% F-media), T2 (50% vitrification media + 50% F-media) and T3 (25% vitrification media + 75% F-media) were used to store PDL cells for 2 weeks and 4 weeks in liquid nitrogen. MTT assay was performed to examine the viability of PDL cells.

**Results:**

Maximum cell viability was achieved in cells stored in 100% conventional cryopreservation media at -196℃ (positive control group) in step 1. Compared to the positive control group, viability of the cells stored in 100% vitrification media was very low as 10% in all test conditions. In step 2, as the percentage of vitrification media decreased, the cell viability increased in cells stored for 2 weeks. In 4-week storage of cells in step 2, higher cell viability was observed in the T2 group than the other vitrification formulae while the positive control group had the highest viability. There was no statistically significant difference in the cell viability of 2-week and 4-week stored cells in the T2 group.

**Conclusions:**

These observations indicate 100% vitrification media is not successful in PDL cell cryopreservation. Conventional cryopreservation media is currently the most appropriate media type for this purpose while T2 media would be interesting to test for long-term storage of PDL cells.

## INTRODUCTION

Preservation of teeth for future use, mainly for autografts and for selected allografts, shows potential for organization of a tooth bank. The proper storage of donor teeth in order to maintain the viability and differentiation capability of periodontal ligament (PDL) cells is an important factor in determining success after autotransplantation. Transplantation of a healthy tooth has been reported to induce the regeneration of the destroyed alveolar bone through the differentiation capability of PDL cells [1].

Cryopreservation is the method of choice for long term storage of living tissues. Despite its disadvantages, this technique has been practiced for many decades in preserving the vital functions of the cells of many types of mammalian and human tissues. A method for cryopreservation of mature teeth has been developed by modifying the techniques used for cryopreservation of mammalian embryos [2]. Successful autotransplantation and allotransplantation of cryopreserved teeth using this technique have been reported [2,3]. The conventional cryopreservation method has been proven to maintain the membrane integrity, viability, and differentiation capability of PDL cells [3-5].

Although the functions of PDL cells are saved by the conventional cryopreservation method, it can cause various types of injuries to the cells during each step of the process. The primary injury is caused by the formation of intracellular ice (ice crystallization) during cooling to -196℃. Secondly, the cryoprotectants that are added to prevent primary injury could cause chemical toxicity and osmotic change, causing swelling of the cells [6].

Tooth cryopreservation should preserve both tooth hard tissue and soft tissues for a successful replantation sparing the masticatory functions. A previous study by Oh et al. [3] reported the occurrence of a longitudinal fracture in 25% of conventionally cryopreserved teeth during a hardness test. Therefore, this study was designed to develop a new freezing method for tooth preservation.

Vitrification, which is defined as the solidification of a solution by an extreme elevation in the viscosity without crystallization, has been developed as an alternative cryopreservation procedure aiming to minimize tissue injuries [6]. A variety of cells, and more recently tissues, have been successfully cryopreserved using vitrification [7-10]. This approach has been applied to improve functional survival of living tissues after freezing and thawing.

Although vitrification has been successfully applied to many cells and tissues such as embryos, monocytes, endothelial cells, vascular grafts, and skin grafts, no attempt has been taken so far to vitrify teeth, tooth buds or PDL cells to the best of our knowledge.

As vitrification has shown cell functional and survival recovery superior to conventional cryopreservation methods, in this study we examined the effects of vitrification on PDL cells. For successful tooth transplantation, the integrity of PDL cells is an utmost need; therefore, the purpose of this study was to evaluate whether vitrification can be useful for tooth cryopreservation. In this study we tested and compared the viability of PDL cells after vitrification or conventional cryopreservation, each for two and four weeks.

## MATERIALS AND METHODS

### Cell isolation and culture

Human PDL tissue was obtained from patients having healthy first premolar teeth extracted for orthodontic purposes. Prior to extraction, informed consent was obtained. The study was approved by the ethics committee of the Yonsei University College of Dentistry.

Each extracted tooth was gently washed with saline and immediately placed in F-medium, which was composed of Dulbecco's modified Eagle's medium (Gibco BRL, Life Technologies, Grand Island, USA) and Ham's nutrient mixture F-12 (Gibco BRL, Life Technologies, Grand Island, USA) in a ratio of 3 to 1, supplemented with 10% fetal bovine serum and antibiotics, consisting of penicillin (100 units/µL), streptomycin (100 µL/mL), and Fungizone (0.3 µg/mL) (Gibco BRL, LifeTechnologies, Grand Island, USA).

PDL was gently separated from the surface of the root by scraping it into a Petri dish with 10 mL F-media. The PDL attached to the middle third of the root was cut from the root surface using a scalpel knife to avoid incorporation of gingival cells. Separated tissue was minced in to tiny pieces less than 0.5 mm^3^. After washing several times in phosphate buffered saline (PBS), the fragments of the PDL were placed as explants in plastic culture flasks with 20 mL of F-media. The culture flasks were incubated at 37℃ in a humidified atmosphere of 5% carbon dioxide to allow the tissue to attach to the walls. The culture medium was not changed until outgrowth of cells was seen. Culture media were then changed every 2nd or 3rd day. At confluence, the fibroblasts were trypsinized and subcultured. PDL cells from passage 6 to passage 8 were used for the experiment to achieve their maximum proliferative potential and homogeneity.

### Preparation of cryopreservation media

The conventional cryopreservation media was prepared by mixing 80% F-medium with 10% fetal bovine serum (FBS) and 10% dimethyl sulfoxide (DMSO) (Me_2_SO; Sigma Chemical, St. Louis, USA). Vitrification media was prepared according to the formula developed by Kasai and Mukaida [6] named ESF40. This comprises 40% ethylene glycol (EG) (WAKO Pure Chemical Industries Ltd., Osaka, Japan), 18% Ficoll 70 (SIGMA, St. Louis, USA), and 0.3 M sucrose (Junsei Chemical Co. Ltd., Tokyo, Japan) dissolved in F-medium.

### Cryopreservation procedure

#### Step 1

As a starting point, 100% vitrification media and 100% conventional media were each used as storage media. The cell lines were grouped by storage conditions as indicated in Table 1. At confluence, tissue culture dishes were washed with PBS 3 times and trypsinized using 1 mL of 0.25% trypsine and 0.08% ethylenediamine tetraacetic acid (EDTA), and harvested in F-media. Five cryotubes were prepared for each experimental group. One mL of resuspended cells was aliquoted into each cryotube, and immediately placed on ice after proper tightening of the caps. For the V3 group, the cryoprotectant was added in two steps, 25% of vitrification media was added at 20℃ and maintained for 10 minutes at the same temperature for equilibrium. Hundred percent vitrification media maintained at 4℃ was added as Step 2. Cell groups V1, V3, and C1 were cooled at approximately -1℃/minute using a dump-freeze method consisting of the suspension of vials in an isopropanol bath within a -85℃ mechanical freezer for 24 hours, followed by plunging into liquid nitrogen (LN_2_) for storage at -196℃. Cell groups V2 and C2 were placed in a separate storage box and stored at -20℃ in freezer. The negative control group was stored at 4℃ in the refrigerator.

Cells were stored for at least two weeks prior to recovery and evaluation. To evaluate the vitality of PDL cells, vials were retrieved from storage and immediately thawed in a 37℃ water bath with an approximate cooling rate of 20℃/minute. On the basis of the results of step 1 we designed step 2 of the experimental protocol.

#### Step 2

According to the literature, we changed the original formula of the vitrification media as indicated in Table 2. The percentage composition of media is indicated in Table 3. All cell groups except the negative control group were cooled at the rate of approximately -1℃/minute using a dump-freeze method consisting of suspension of vials in an isopropanol bath within a -85℃ mechanical freezer for 24 hours, followed by plunging into LN_2_ for storage at -196℃. The negative control group was stored at 4℃ in the refrigerator. The storage cell retrieval method was similar to that of step 1. Cell retrieval was done after 2 weeks and 4 weeks of cryopreservation.

### MTT assay

MTT assay was carried out to check the viability of PDL cells preserved in different storage conditions. Each experiment was repeated at least three times. Cell lines from all groups were immediately thawed in a 37℃ water bath and the cells were harvested in F-media at 4,000 rpm for 4 minutes. A 96-well tissue culture plate (Microtest™ 96, BD, Franklin Lakes, USA) was plated with 150 µL of F-media, containing 2 × 10^4^ cells/well. After overnight incubation of the samples at 37℃, the remaining media was removed by aspiration. 150 µL/well of yellow MTT solution (MTT: 3-[4,5-dimethylthiazol-2-yl]-2,5-diphenyl-tetrazolium bromide at 0.05 mg/mL; Sigma Chemical, St. Louis, USA) was added to each well. The plates were incubated at 37℃, in a humidified atmosphere with 5% carbon dioxide for 3 hours. The remaining untransformed MTT in the supernatant was then removed by aspiration. The formazan crystals were dissolved by the addition of 150 µL/well of DMSO (Sigma Chemical, St. Louis, USA). After a few minutes, 80 µL/well dissolved formazan in DMSO was transferred to another 96 well plate. The plates were then placed into an enzyme-linked immunosorbent assay reader (ELISA reader; Benchmark Micro plate reader, Bio-Rad, Hercules, USA) and their optical densities read at a wavelength of 570 nm to measure the maximal absorbance of the solubilized formazan product. For each group, the mean optical density value was calculated.

### Morphology of test groups after 2-week cryopreservation

One set of stock cell vials in the step 2 group was used to assess the morphology of PDL cells after 2 weeks of storage. After 2 weeks of storage, the cryovials were thawed as described for the viability assay. 2 × 10^4^ cells/mL were seeded in 60 mm dishes in F-media and incubated at 37℃ in a humidified atmosphere with 5% carbon dioxide. Media were changed on the following day. Low magnification photographs of cell growth were taken on day 7.

## RESULTS

### Viability of PDL cells in step 1

**Figure 1:**
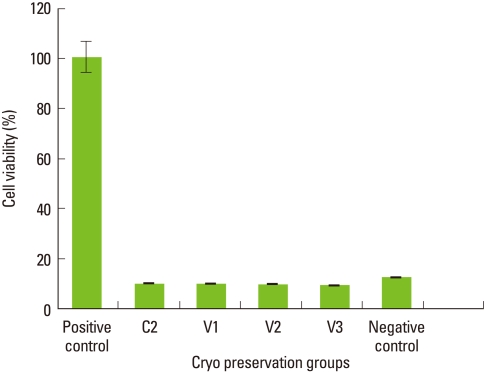
The viability of periodontal ligament cells by different storage conditions in step 1. Mean values normalized using the positive control. Columns and bars represent the mean of three independent experiments±SD.

The results of the MTT cell viability test for step 1 is shown in Fig. 1. According to the results in step 1, the positive control group or the conventional cryopreservation group stored at -196℃ showed maximum cell viability while all the other groups showed much decreased cell survival closer to 10% when compared to the positive control group.

### Viability of PDL cells in step 2

#### Cryopreservation for 2 weeks

**Figure 2:**
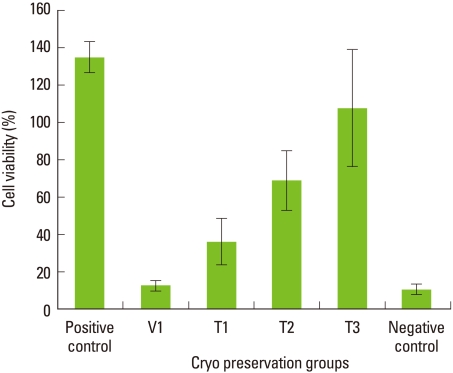
The viability of periodontal ligament cells after 2-week preservation in step 2. Mean values normalized using the positive control. Columns and bars represent the mean of three independent experiments±SD. As the percentage of vitrification media decreases, the cell viability was inversely increased. The T2 group showed around 75% cell viability compared to the positive control. There was no statistically significant difference between the positive control and T3 groups (*P*>0.05).

Average cell viability of cells stored for 2 weeks in the step 2 test is shown in Fig. 2. These results also indicate a very low cell survival rate in 100% vitrification media. As the percentage of vitrification media decreases, the cell viability was inversely increased. The difference in cell viability within cell groups is statistically significant (*P*<0.05; Using ANOVA). The T3 group showed the highest cell viability in this group, but statistically, there was no significant difference in cell viability between the positive control group and T3 group (*P*>0.05). When compared to the positive control, the T2 group had around 80% cell viability, while the vitrification media-only group showed the lowest cell viability to be around 10%. There was a statistically significant difference between the positive control and T2 groups (*P*<0.05). Statistically, there was also a significant difference between the T2 and T3 groups (*P*<0.05). Also, a statistically significant difference was seen between the T1 and T3 groups (*P*<0.05).

#### Cryopreservation for 4 weeks

**Figure 3:**
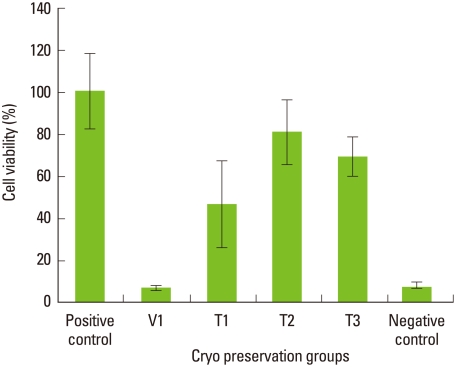
The viability of periodontal ligament cells after 4-week preservation in step 2. Mean values normalized using the positive control. Columns and bars represent the mean of three independent experiments±SD. The T2 group shows around 80% cell viability compared to the positive control. There is no statistically significant difference in the viability of T2 group cells stored for 2 weeks vs. 4 weeks (*P*>0.05). Viability of T3 group cells had reduced in 4-week storage compared to 2-week storage, and the reduction was statistically significant (*P*<0.05).

Average cell viability of cells stored for 4 weeks in the step 2 test is shown in Fig. 3. The difference in cell viability among the cell groups is statistically significant (*P*<0.05; Using ANOVA). Interestingly, the T3 group showed much reduced cell viability. Statistically, there was a significant difference in cell viability between the positive control group and T3 groups (*P*<0.05). When compared to the positive control, the T2 group had around 80% cell viability, while the vitrification mediaonly group showed the lowest cell viability to be around 10%. There was a significant difference between the positive control and T2 groups (*P*<0.05). Statistically there was a significant difference between the T2 and T3 groups (*P*<0.05). Also, a statistically significant difference was seen between the T1 and T3 groups (*P*<0.05).

The average cell viability of the T2 and T3 groups after 2-week storage and 4-week storage was statistically compared. Statistically, there was no difference in cell viability between 2-week storage and 4-week storage of T2 group cells (*P*>0.05). There was a statistically significant difference of cell viability between the 2-week storage and 4-week storage of T3 group cells (*P*<0.05).

### Morphology of test groups of step 2 cultured after 2 weeks of storage at different conditions

**Figure 4:**
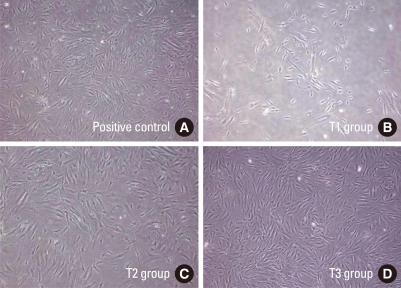
Morphology of periodontal ligament cells after two-week storage in step 2. After two-week storage, cryopreserved vials were thawed and 2 × 10^4^ cells/mL were subcultured in F-media. Light microscopic photographs were taken at day 7. In all groups, the cultured cells showed a spindle-shaped morphology. Compared to the positive control (A), the T1 (B) group showed a paucity of viable cells.

Low magnification photographs of cell growth were taken on day 7 (Fig. 4). PDL cells showed a spindle shaped and elongated appearance. After 2-3 days of subculture they had adhered well and uniformly spread growth was seen under light microscopy. There was no detectable morphologic difference seen among the positive control and the T2 and T3 group cells.

## DISCUSSION

Vitrification is the newest cryopreservation method practiced in cryobiology and is reported to be successful in various fields for a variety of cells and tissues [7-10]. The term vitrification-literally 'turned into glass' - is defined as the conversion of a system from a fluid to a solid solely by an increase in viscosity, without a phase-change, without any crystallization of water, and therefore in the complete absence of ice [11].

Many vitrification profiles have been developed and practiced on various cells and tissues including human and animal embryos. There are many ongoing experiments in this field towards a better success rate. As this new method has gained success in many aspects we thought of applying it to preserving tooth tissue. PDL cells were selected as the group to be tested as a preliminary study because PDL cell survival is crucial in tooth cryobiology.

The aim was to compare the viability of stock cells stored in these two different cryoprofiles, namely the vitrification method and conventional method at a temperature of -196℃. Cells stocked in conventional media stored at -196℃ were used as the positive control. Cells stocked in conventional media stored at 4℃ were used as the negative control. In addition, we compared the viability of cells stored at -20℃ in both media to simulate the available storage conditions in a general dental practice, to study whether in an emergency situation is it possible the tooth could be initially stored in the same medium as that in which it is preserved for the long term.

To vitrify tissues at practicable cooling rates requires exposure to high, multimolar concentrations of solutes that readily form glasses. Typically, vitrification solutions comprise complex mixtures of solutes on the basis that none of the individual components exceeds its putative toxic concentration, yet the overall solute concentration is sufficient to vitrify at practicable cooling rates [12].

The vitrification medium mainly consists of a permeating cryoprotectant, which is the essential component, for example, EG. Usually this is supplemented with a macromolecule, like Ficoll 70 and a small molecule, like sucrose. Various formulae have been developed using these basic components for different cell types [6].

A permeating agent is an essential component in all vitrification solutions. There are 5 different cryoprotective permeating agents widely used, namely: DMSO, acetamide, propylene glycol, glycerol, and EG. Either used in combination or as a single permeating component, the mechanism of the protective properties of the above agents is considered the same, but the toxicity and the permeating properties of the agents are quite different. Among the five agents, EG and glycerol are less toxic than DMSO and propylene glycol, and acetamide is the most toxic when used in cryopreservation of embryos [6].

Macromolecules are non-permeating to the cell and much less toxic than permeating agents. By incorporating a macromolecule, it should be possible to reduce the toxicity of the solution by decreasing the concentration of permeating agents required for vitrification of the solution. At this point, Ficoll 70 seems to have the advantages of lower toxicity, higher solubility, and lower viscosity [13].

Macromolecules contribute to the osmolarity of the solution only a little, while mono- or disaccharides exert considerable osmotic effects as smaller non-permeating molecules. In fact, it was found that incorporation of sucrose reduced the toxicity of a vitrification solution significantly [13].

The formula we used here has often been used to vitrify human and animal embryos. We used this as the base formula because the current conventional cryopreservation formula is also a modification of embryo preservation formula. Although vitrification is found to be effective in cryopreservation because it prevents ice crystallization, thus preventing cellular injury, on the other hand, the cryoprotectants which are used in high concentrations are themselves toxic to the cells. Even though a combination of macromolecules is used to reduce this risk, they are not proven to have no substantial adverse effects. The best cryoprotectant for a given solution will depend on the specific cell and on a range of considerations including the presentation of cells--either isolated or stagnated as a tissue, the nature and concentration of the cryoprotectant, and the temperature at which it is added, the rates of cooling and warming, the storage temperature, and the temperature and rate at which the cryoprotectant is removed [11,14]. The crucial points in using cryoprotectants in high concentration are that there is always a toxic limit to the concentration of cryoprotectant that can be used. The increased osmolarity of the solution could cause osmotic shock to the cells resulting in cell damage and ultimately cell lysis [14].

Even though the vitrification technique had changed the cryopreservation profiles in reproductive medicine, its use is controversial. Although vitrification is useful in oocyte and embryo preservation, conventional freezing is more promising in cryopreservation of human ovarian tissue because of its high developmental potential [15]. The results based on the investigations done on human ovarian tissue have shown conventional freezing is more effective in terms of gene stability [15]. Kasai and Mukaida [6] and Isachenko et al. [15] summarized the possible reasons for the low survival of embryos after vitrification as (a) sensitivity of embryos to chilling (b) lower permeability of the cell membrane leading to intracellular ice formation and osmotic over-swelling, and (c) toxicity of the cryoprotectant during exposure of cells to the medium. Attempts to vitrify corneas in these multicomponant vitrification solutions have also been unsuccessful [16,17]. For corneal vitrification, a very high concentration of cryoprotectants had to be used and during warming it caused corneal endothelial damage [18].

We attempted to evaluate whether PDL cell vitrification can be useful for tooth cryopreservation. 100% ESF40 media (V1 media) with very high levels of cryoprotectants might have caused damage to PDL cells by the toxicity of cryoprotectants itself and by osmotic shock. The first reason for the low survival could have been the sensitivity of cells to the high level and toxic effects of the cryoprotectants used. High osmotic concentration of the solution might have acted as the second reason. To overcome the cellular injuries caused by these reasons, modified vitrification methods have been developed with lower concentrations of cryoprotectants, making a less toxic solution. The effectiveness of this approach has been reported by many researchers [6].

In step 2, the vitrification medium was diluted as illustrated in Table 2. As the concentration and the osmolarity were reduced, the percentage of cell survival increased accordingly. Cells were retrieved after 2-week and 4-week storage. In 2-week storage, the T3 group showed the highest viability among all groups but there was no statistically significant difference between the positive control and the T3 group (*P*>0.05). Compared to the positive control, the T2 group displayed an approximately 80% cell survival rate, with similar cell morphology to the positive control group cells when subcultured after two-week storage. Cell morphology of the T3 group was also similar to that of the positive control group. There was a statistically significant difference in cell viability between the T2 and T3 groups (*P*>0.05).

In 4-week storage, the T3 group showed a slightly reduced cell viability compared to the T2 group, and the difference was statistically significant (*P*<0.05). The positive control group showed the highest cell viability among all groups. The T2 group displayed an approximately 80% cell survival rate, and there was a statistically significant difference between the positive control and T2 groups (*P*<0.05).The difference between the T3 group and the positive control groups was also statistically significant (*P*<0.05). Cell viability of the T2 and T3 groups were each compared between 2 week storage and 4 week storage. There was no significant difference in the viability of cells in the 2-week storage and 4-week storage of the T2 group (*P*>0.05). In the T3 group there was a statistically significant difference in the viability of cells in the 2-week storage and 4-week storage (*P*<0.05). Although the cell viability of the T2 group was likely to be 80% of that of the positive control group, it could maintain a similar viability level at 2-week storage and 4-week storage. Therefore T2 storage medium would be interesting to study further.

EG has been considered superior to DMSO as a cryoprotectant because of its lower toxicity to cells [6,11,19]. In previous studies where they have been tested among combinations of EG + DMSO and EG + Ficoll 70 it has been shown that Ficoll 70 was more effective than DMSO when combined with EG [19]. When EG has been used in combination with DMSO in rabbit embryo preservation, severe subcellular damage and osmotic damages causing rupture of the plasma membrane have been reported, and a better post-thaw survival of embryos has been achieved by using a combination of EG and Ficoll 70 [19]. In T2 medium we have used a combination of EG and Ficoll 70 based in F-media. The percentage concentrations of these ingredients were kept at 50% of that of the widely used vitrification medium. By means of this, the toxicity of these cryoprotectants and the resulting osmotic damage would have been reduced. Therefore, the combination of T2 media would be useful for the future studies.

Up to now conventional cryopreservation and autotransplantation of teeth have been successful. Physical properties of tooth hard tissue of these frozen teeth should be closely assessed. Our results also favor conventional cryopreservation media as the best for PDL cell storage at -196℃. If the long term PDL survival rate in T2 media is closer to that of conventional media, whole tooth preservation could be tested using T2 media, focusing on the maintenance of the physical properties of tooth hard tissue.

## Figures and Tables

**Table 1:**
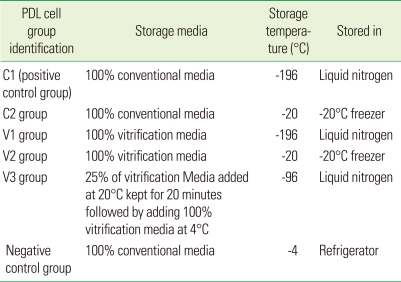
Cell grouping in step 1 according to storage conditions.

PDL: periodontal ligament.

**Table 2:**
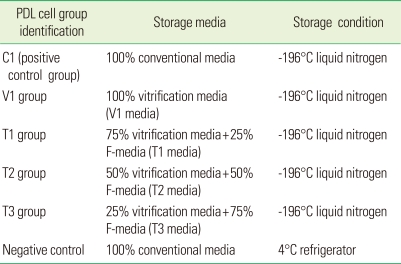
Cell grouping in step 2 according to storage media and storage conditions.

PDL: periodontal ligament.

**Table 3:**
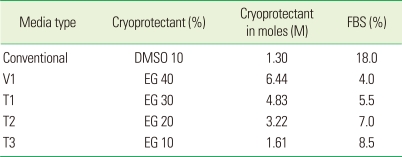
Composition of media in step 2 cell grouping.

Conventional: 100% conventional cryopreservation media, V1: 100% vitrification media, T1: 75% vitrification media + 25% F-media, T2: 50% vitrification media + 50% F-media, T3: 25% vitrification media + 75% F-media, FBS: fetal bovine serum, DMSO: dimethyl sulfoxide, EG: ethylene glycol.
